# Social Media Friends From Afterschool are Associated With Positive Youth Development in Digital Settings

**DOI:** 10.5195/jyd.2022.1173

**Published:** 2022

**Authors:** Lisette M. DeSouza, Linda Charmaraman, Emily Vargas, Georgia S. Hall

**Affiliations:** Wellesley Centers for Women, Wellesley College

**Keywords:** after-school activities, middle school, positive youth development, peers, social media, adolescence

## Abstract

Positive youth development has been extensively documented in contexts such as the family, school, and afterschool. Emerging theory and research indicate that digital contexts such as social media may also be venues through which young people develop skills and attributes associated with the 5 Cs model of positive youth development and thriving. This research attempted to understand if and how middle school youth’s in-person and online networks connect, and if they do connect, do these connections relate to engaging in beliefs and behaviors associated with PYD. Results suggest that in this sample, middle school youth include peers from afterschool in their online networks, and those who have friends from afterschool and school engaged in PYD- related social media behaviors at higher rates than those who were not connected to in-person networks. No association was found between the amount of time spent in after-school contexts and any of the positive or problematic social media outcomes in this study. Implications for youth development professionals considering the influence of social media on youth, and next steps for research on after-school activities and social media use of middle school youth are discussed.

## Youth Activity Participation and Social Media Use

In the United States, 7.8 million children (13%) were enrolled in after-school programs (Afterschool Alliance, 2020), 90% of teens used social media (Rideout & Fox, 2018), and approximately 73% of 10- to 12-year-old adolescents had signed up for social media sites despite age restrictions limiting use before age 13 (Coughlan, 2016). These indicators suggest that both afterschool and social media are important contexts in the lives of youth. The question remains, however, how do youth relationships with their peers from afterschool connect to experiences online? Research has demonstrated that youth participation in organized activities, like after-school programs, can shape overall well-being, peer networks, and burgeoning identities (Durlak et al., 2010; Vandell et al., 2015). These outcomes are particularly important during early adolescence and in middle school. Emerging research demonstrated that social media also plays a supportive role in young adolescents’ social and emotional well-being, influences peer networks, and informs their overall identity development (Charmaraman et al., 2020), despite a tendency for research and practice to focus on the risks of social media (Ross & Tolan, 2021). Therefore, the present research capitalizes on a secondary data set to link youth in-person after-school peers with their experiences online.

Although research has indicated the importance and salience of both contexts on youth development, little is known about how in-person experiences in after-school activities relate to youth’s experiences online. In the present research we explore if and how youth peers and participation in afterschool is associated with the social media experiences of (a) frequency of checking social media, (b) cyberbullying, (c) seeking peer support, and (d) providing peer support.

### Social and Emotional Developmental Experiences During Early Adolescence

During adolescence, youth are undergoing cognitive, social, and emotional changes. They become more peer-oriented than in earlier developmental periods; they look for opportunities to lead and be autonomous; and they develop identities in multiple domains. Younger adolescents (i.e., youth in ages 11 to 14) are particularly aware of their peer status and self-esteem becomes more consequential in this developmental period than in other developmental periods (Brinthaupt & Lipka, 2012), so it is important to pay particular attention to the social and emotional needs and experiences of youth during this period.

For these young adolescents, positive behaviors might include developing prosocial peer relationships, feelings of competence, and establishing a sense of self (Steinberg, 2007). Conversely, increasing autonomy and a focus on peers might also relate to problematic social situations such as bullying, or a negative self-concept. Positive and problematic behaviors can coexist, and manifest in diverse ways across youth experiences and in the varied domains that young people inhabit - in schools, in families, with peers, online, and in after-school programs.

### A Positive Youth Development Perspective on Afterschool and Social Media

A positive youth development (PYD) perspective suggests that when the strengths of youth are aligned with the assets in their contexts, thriving occurs. PYD has been extensively documented through research about youth development and after-school programs (Geldhof et al., 2014; Smith et al., 2017). In the Five Cs model, positive development is operationalized as competence, confidence, connection, caring, and character (Lerner, 2004). When young people exhibit the Five Cs, they contribute positively to themselves, to others, and to civil society (Lerner, 2004). Ross and Tolan (2021) suggest that digital contexts such as social media also have the potential to be PYD contexts. They indicate that because PYD is focused on this alignment between youth strengths and the context, the digital setting is a context “brimming with opportunity for positive interactions to occur” (Ross & Tolan, 2021, p. 532), and that “digital media shares many of the same characteristics of traditional PYD settings that make them ideal for positive development to occur (e.g., permanence, engaging, interactive, collaborative, and mutually beneficial)” (p. 535). In particular, Ross and Tolan highlight that young people can build their social emotional skills and relationships online. Although measures do not currently exist to capture PYD in digital spaces, it is possible that the Cs can be operationalized in digital settings (Ross & Tolan 2021). For example, youth can demonstrate caring by engaging empathetically with others online (Ross & Tolan, 2021). In the present research we employed the PYD perspective and in particular the relational aspects of caring and connection conceptualized within the Five Cs model to understand the links between youth’s in-person and online interactions.

### After-School Activities Shape Social and Emotional Outcomes

After-school activities can help adolescents develop the skills associated with positive peer relationships and a healthy sense of self. Research has shown many positive outcomes associated with high-quality activity participation for youth. For example, in a meta-analysis of 68 studies of after-school programs (37% serving middle school youth) that seek to promote personal and social skills in youth, researchers found an association between youth attending after-school programs and increased positive feelings about themselves and their schools, and increased positive social behaviors (Durlak et al., 2010). Russel et al. (2006) reported that middle school programs provided opportunities for identity formation and leadership development which can be helpful to address the transition from elementary to middle school. Russell et al. concluded that after-school programs play an important role in developing youths’ social skills and provide a space less demanding than the school day where youth have opportunities to form and sustain relationships.

This overview of research associated with activity participation suggests that after-school contexts have the potential to promote social development (e.g., developing relationships, social bonding) and emotional development (e.g., positive feelings about oneself, identity development). Next, we review youth social media use and how social media engagement might also shape social and emotional development for youth.

### Social Media Experiences

Like after-school activities, social media use can be related to prosocial behavior within online communities. Despite the perception of mostly negative online experiences, only a quarter of teens (24%) describe social media as a primarily negative experience (Anderson & Jiang, 2018a). In fact, in nationally representative studies of teens aged 13-17 using social media (Anderson & Jiang, 2018b), 81% felt more connected to their friends and 68% of online friends supported them through tough times through social media. There are ways that teens interact online which may shape their social behaviors, and the majority of teens used the Internet as a healthy venue for social interaction (Lenhart et al., 2010). For example, frequent online communication was associated with higher quality friendships (Valkenburg & Peter, 2007), and frequent phone use, email, and messaging is related to increased social competence (Ohannesian, 2014). Adolescents who are doing well socially may also benefit from online activity because it supports the formation of friendships (Kraut et al., 2002; Peter et al., 2005). For example, the stimulation hypothesis suggests that the time that youth spend online involves intimate disclosures which enhances closer friendships (Alison Bryant et al., 2006; Valkenburg & Peter, 2007). Furthermore, interactions through texting and social media have been found to promote a sense of belonging (Davis, 2012). This online communication has also been found to facilitate better self-concept clarity across social situations (Davis, 2013). Finally, social media can improve adolescent social connectivity and sense of belonging as well as provide a forum for self-disclosure and identity exploration (James et al., 2017). This research suggests that rates of problematic experiences online are relatively low, and that the majority of youth are more likely to have positive emotional (e.g., self-concept) and social (e.g., quality friendships) developmental experiences.

It is important to acknowledge that like any context in the lives of youth, there is the potential for less desirable youth behaviors and interactions amidst the potential for positive development (Ross & Tolan, 2021). According to Common Sense Media (Rideout & Robb, 2019), 8- to 12-year-old youth spent an average of 4 hours and 44 minutes per day on screen media which increases with age, such that 13- to 18-year-old youth spend an average of 7 hours and 22 minutes online per day. Given the average amount of time spent on social media per day, youth often spend more time using digital media than participating in after-school or other supervised programming. Young people’s use of social media can span the spectrum of positive to problematic behavior. In a recent meta-analysis of cyberbullying victimization, studies varied quite widely in prevalence estimates (3% to 72%) due to inconsistent definitions and time periods assessed (Selkie et al., 2017). Pew Research (Anderson, 2018) has demonstrated that the more frequently teens are online, the more likely they are to be cyberbullied. Cyberbullying and online victimization has also been related to student experiences in school such as number of detentions, suspensions, and truancy (Ybarra et al., 2005). When measuring recency and frequency, the rate of cyberbullying was rather low (especially compared to in-person forms of bullying). In a population-based sample of 120,000, only one in 20 girls and one in 50 boys experienced cyberbullying (Przybylski & Bowes, 2017). Despite these inconsistent and relatively low rates of cyberbullying, it remains important to document if and how the potential for this risk may exist in the lives of middle school youth.

### Connecting Afterschool With Social Media

The research described above suggested that high quality after-school programs can contribute to positive social and emotional outcomes such as better self-esteem, school bonding, and prosocial behaviors (Durlak et al., 2010). Notably, high participation in these programs has been found to influence prosocial behavior (Fredricks & Eccles, 2008). Social media also has the potential to contribute to young peoples’ social connectivity and identity development (James et al., 2017). The structures, philosophies, and strategies of after-school activities (e.g., the expectations for positive peer relations) may reinforce standards for positive and supportive interactions on social media, and “in-person” and virtual friends might connect across these two domains. Pragmatically, it is also possible that being engaged in an after-school activity limits the amount of time that a young person has available to check and participate in social media. An alternative is that engagement in after-school activities may facilitate online connections with friends beyond in-person experiences and lead to increased interest in engaging online.

Despite the potential for positive engagement in both domains, and in particular the potential for social media to deepen relationships which started in person, research has not yet linked these two domains. In the present research, we were interested in two indicators of after-school participation and their associations with youth online engagement. First, given that high participation is related to “in-person” prosocial behavior (e.g., Fredricks & Eccles, 2008) we incorporated the amount of time that youth spend in after-school programs to assess whether this type of “dosage” either limits online engagement or enhances youth social and emotional skills. Second, we examined whether youth are connected to their “in-person” peers from afterschool as “friends” online. Given the potential for both positive and problem behavior in the online space, our hypothesis about this indicator was open-ended. Overall, we hypothesized that being a part of after-school activities may be related to more supportive online behaviors due to possible scaffolding that may occur in these programs which typically offer mentoring and structured peer interactions (Eccles & Gootman, 2002). In particular, we focused on outcomes related to seeking support (connecting) and providing support (caring) via social media. Acknowledging the potential for risks online we examined the links between these after-school indicators and experiencing negativity online. Given the application of a PYD theoretical framework, and existing research documenting the positive and prosocial ways that youth engage online, we expected a positive association between after-school indicators (i.e., social media friends from afterschool, and time in afterschool) with the PYD-focused outcomes, and a negative association with the risky outcome (i.e., experiencing negativity).

## Method

### Procedure

As part of an ongoing longitudinal study of the social contexts and behavioral consequences of adolescent social technology use (Charmaraman et al., 2022; Charmaraman et al., 2020; Charmaraman et al., 2021), middle schools in the Northeastern United States were recruited in 2019 based on varying school enrollment size, Internet accessibility, and diverse racial/ethnic composition. Sites were also selected that had robust after-school and/or sports activity offerings. Upon obtaining approval from the Wellesley College Institutional Review Board, and institution and school district-level permissions, we worked with liaisons in one school and in one district-wide after-school program spanning three schools to distribute informed consent/opt-out forms (in English, Spanish, and Portuguese) to parents through paper flyers, parent email listservs, school e-newsletters, and direct emails. For the Fall 2019 data collection, members of the research team proctored the online Qualtrics survey in person during a prescheduled advisory period or after-school program break lasting up to 60 minutes. Youth primarily used school or after-school program-provided Chromebooks and the survey took about 25-40 minutes to complete. Because survey links were emailed to youth through their Google Classroom or after-school program listserv, those who were absent during the survey administration were able to participate from home.

An honorarium was provided to schools for participating as well as gift card incentives to teachers and program coordinators to help with survey recruitment. Youth who participated in the survey were given an embossed pen, snacks (in after-school programming), and were entered into a drawing prize for a chance to win a $25 gift card.

### Participants

Participants (*N* = 1033) in the larger study were in fifth through ninth grades, 49% female, 49% male, and 2% other. Self-reported race was 7% Asian, 10% Black, 19% Latinx, and 48% White. Twenty-three percent of youth received free or reduced-price lunch and 77% did not receive or did not know if they received free or reduced-price lunch.

The sample for the present analyses included youth in middle school (Grades 6 through 8) who also responded to the time in activities indicators described below. This selection resulted in 687 participants: 33% sixth, 34% seventh, 34% eighth grade. Ninety-three percent of youth participated in an “after-school” or “sport” based activity. Youth described their gender as 50% female, 48% male, and 2% other. Youth reported their race as 8% Asian, 14% Black, 32% Latinx, 5% Native, 52% White. Twenty-four percent of youth received free or reduced-price lunch, 44% of youth did not receive free or reduced-price lunch, and 33% did not know.

### Measures

#### Checking Social Media

To indicate how often youth check social media, we asked: “On a typical school week, how often do you check social media (like Instagram)?” Responses were 1 (*never/does not apply to me*), 2 (*every few days*), 3 (*once a day*), 4 (*every few hours*), 5 (*every hour*), 6 (*more than every hour*). The average score for checking social media was 3.50 (*SD* = 1.92).

#### Experiencing Negativity Online (Risks)

Experiencing negativity was measured with an average of three items adapted from a scale of Internet harassment developed by Ybarra and colleagues (2007). Youth responded to three items requesting how often a certain online experience happened to them: “You were hurt by someone making you feel left out online,” “Someone made a rude or mean comment to you online or by text,” and “You were hurt by someone making you feel left out online (including social media, games)” on a scale of 1 (*Never*) to 5 (*Always*). The average score for was 1.40 (*SD* = .54) and *a* = .77.

#### Giving Support (Caring)

Youth were asked how often they: “post something to make others feel good,” “respond positively when friends share good news,” and “try to make friends feel better when they share bad news.” These indicators were developed by the project team. Responses ranged from 1 (*Never*) to 5 (*Always*). The average score for giving support was 3.27 (*SD* = 1.14) and a = .82.

#### Online Support Seeking Behaviors (Connection)

Support seeking behaviors on social media were measured with an average score of five items, adapted from the Facebook Measure of Social Support (McCloskey, 2015), using subscales Perceived Support and Negative Social Support. Youth responded to five items: “When I am stressed out, I turn to my friends for help on this site,” “The support I get on this site makes me feel better,” “When I have a need that friends ignore on this site, I’m hurt,” “This site makes me feel close to people,” and “It’s important that my friends like or comment on my posts.” Responses were on a scale of 1 (*Strongly Disagree*) to 5 (*Strongly Agree*). The average score for seeking support was 2.27 (*SD* = .93) and a = .85.

#### Social Media Friends From Afterschool

Youth were asked “Who are your friends (or who do you follow) on social media (Instagram, etc.)?” If they selected “Friends from after-school activity or team” they were coded as (1) and compared with youth who did not select this category (0). Fifty-three percent of youth in the present sample were social media friends with peers from their after-school activities.

#### Social Media Friends From School

Youth were asked “Who are your friends (or who do you follow) on social media (Instagram, etc.)?” If they selected “Classmates in upper grade” they were coded as (1) and compared with youth who did not select this category (0). Similarly, if they selected “Classmates in your grade or younger” they were coded as (1) and compared with youth who did not select this category (0). Forty-five percent of youth reported friends who were older classmates and 59% of youth reported same or younger age classmates.

#### Time in Afterschool

Youth responded to the prompt: “During a typical 5-day school week, what do you do after school? Indicate how many days a week you do the following: Attend an after-school program or club (e.g., drama).” Responses were in *number of school days* and ranged from 0 to 5. The average youth spent 1.71 (*SD* = 1.48) days in an “after-school program.” We recognize that the term after-school program to youth could mean something different than after-school program as described by researchers and practitioners, however we use this language here because of relevance to ease of understanding for youth.

#### Demographics

Three demographic indicators were included as covariates in the present analyses. Two gender categories were assessed, female (1) youth were compared to male (0) youth. If youth responses to their gender did not correspond to one of these two options, they were included in the analyses, but their specific gender effects were not sufficiently powered to detect effects in the present analyses. Four race categories were assessed: Black and Latinx and Asian youth were compared to White youth (0). Free or reduced-price lunch participation was used as a proxy for income. Youth who received free or reduced-price lunch (1) were compared to a combined group of youth who were unsure or who did not receive free and reduced-price lunch (0). Finally, youth grade in middle school was assessed continuously: sixth grade (6), seventh grade (7), and eighth grade (8). Demographics and descriptive statistics for all indicators are included in Tables 1-4.

### Analysis Plan

The purpose of this analysis was to test the hypothesis that there is an association between after-school experiences in terms of time spent in afterschool and having social media friends (SM friends) from afterschool, with youth PYD-related experiences online. Using linear regressions conducted in SPSS (v26), we tested the relationship between after-school experiences (i.e., time in afterschool, having a SM friend from afterschool), and four outcomes. PYD-related outcomes included providing support and seeking support. The risky outcome included experiencing negativity. Checking social media was included as an outcome which could span the categorization of positive or problematic. Covariates were included in this model to account for potential differences in background: race, gender, income, and grade level. Social media friends from school were also included as covariates in order to understand the potential for after-school SM friendships to have a unique role in the way that youth typically interact online.

## Results

### Checking Social Media

As shown in Table 1, linear regression suggested that having each type of friend on social media was related to increased checking of social media compared to the group of youth who did not indicate having that type of friend on social media: same/younger age classmates (*B* = .91, *SE* = .18, β = .24, *p* < .001), older classmates (*B* = 1.00, *SE* = .18, β = .26 *p* < .001), and after-school (*B* = .93, *SE* = .16, β =.24 *p* < .001). The amount of time in afterschool was not related to checking SM. In this model, youth enrolled in the free/reduced-price lunch program were also more likely to check social media than those who did not report participation in this program.

### Seeking Support Online

Having SM friends from afterschool (*B* = .27, *SE* = .10, β = .14, *p* = .006), and having SM friends who were older classmates were both positively associated with seeking support (*B* = .35, *SE* = .11, β = .19, *p* = .001) when compared with youth who did not report having social media friends from these groups (see Table 2). The amount of time in afterschool was not related to seeking support online. Youth who reported being female and were enrolled in the free/reduced-price lunch program also were more likely to seek support online than male youth and youth who did not report enrollment in free and reduced-price lunch, respectively.

### Providing Support Online

Having SM friends from afterschool (*B* = .38, *SE* = .11, β =.17, *p* = .001), and SM friends in the same/younger grade (*B* = .45, *SE* = .13, β = .19, *p* = .001) were associated with providing more support online when compared with youth who did not report these types of social media friends (see Table 3). The amount of time in afterschool was not associated with providing support online.

### Experiencing Negativity Online

The only indicator associated with experiencing negativity online was having older classmates as SM friends (*B* = .19, *SE*=08, β = .15, *p* = .020) compared with those who did not report this type of SM friend (See Table 4). Neither after-school indicator (SM Friends nor time in afterschool) was related to experiencing negativity.

## Discussion

The purpose of this exploratory research was to assess the relationship between after-school participation and PYD experiences online. Research has not yet assessed the relationship between these two realms of influence in the lives of youth. The present study showed preliminary evidence that there is a link between youth after-school connections and online experiences, in terms of the peers (i.e., SM friends) that span both contexts. Despite this connection, few after-school programs offer guidance on prosocial behaviors in the digital worlds of youth, recognizing the need for positive reinforcement of social technology use (James, 2013).

Prior research has found that frequent checking of social media and online communication is related to both positive and negative online influences (Charmaraman et al., 2018), so this outcome was assessed to better understand the context of social media use. Youth in after-school programs were checking social media more frequently, which can be either an indicator of feeling connected to one’s supportive online networks or the need to browse passively without connecting. The frequency of youth in afterschool checking social media could be due to the fact that increased network size and diversity of contacts would result in more activity to check. Other research has also found that after-school programs use social media to communicate directly with youth (e.g., Ram Lee & Horsley, 2017). In these cases, youth may be checking social media more frequently to keep up with information and interactions from their after-school programs, which informally extends the offline interactions into the online worlds.

We found partial support for the hypothesis that after-school experiences would be associated with PYD exhibited by youth online—time in afterschool was not related to PYD online, but friends from afterschool did relate to indicators of PYD online. The finding that the amount of time in afterschool did not relate to any of the tested positive or problematic indicators was contrary to our expectations. This finding suggests that to understand the experiences of youth on social media in terms of afterschool, future research should look beyond solely participation in programming. Anecdotally, afterschool may be considered a way to limit the time youth spend online. However, this idea does not bear out in the current data, with more evidence pointing to the relationships and connections from afterschool being extended in the online environment. Furthermore, it is possible that social media use might be concurrent to attendance in afterschool, and the “boundaries of experiencing being on media and being ‘in person’ are disintegrating” (Ross & Tolan, 2021, p. 533).

The peer relationship-based component of afterschool was related to each of the PYD- related outcomes in the present study. There was a positive relationship between having a SM friend from afterschool with seeking support online (connection), and with providing support online (caring). The association between having SM friends from afterschool and school with both seeking and providing support is consistent with research which suggests that youth who are doing well socially might also be doing well online (Peter et al., 2005), and that social media might be a way for youth to extend and deepen their friendships (Alison Bryant et al., 2006; Valkenburg & Peter, 2007). It is also notable that the present research did not find evidence for experiencing negativity online in relation to having SM friends from afterschool. Rather, the evidence suggests that older classmates outside the after-school environment may contribute to negatively perceived online experiences, which suggests that having older classmates may be a risk factor to consider when counseling youth about their online networks. This finding may, in part, alleviate some protectionist concerns that when youth leave the supervision of an after-school space, problem behaviors will occur. Our suggestions for future research about after-school would include a focus on how variation in program practices such as a facilitated supportive environment, and the establishment of peer and community norms within and beyond programming might also shape PYD-related outcomes online. Addition of these types of after-school experiences may also illuminate the non-significant finding related to the amount of time in afterschool. Namely, it might not be only the amount of time in that program, but the quality of time spent in an after-school program, including the ways that quality programs facilitate development of relationships in after-school programs (e.g., Durlak et al., 2010).

### Limitations and Future Directions

These findings should be interpreted in light of a few limitations. This research capitalized on an existing dataset to explore how two after-school experiences—amount of time in program and friends from afterschool—might relate to social media experiences for middle school youth. After-school programming also varies widely in focus, content, and structure, and similarly might result in different social media experiences. Future research should explore specific types of programming and their relationship with youth experiences on social media. In addition, although this sample is diverse in terms of demographic indicators, they engaged in problem behaviors at similarly low rates as those in their age group and participated in programs at high rates. Future research should assess the relationship between activity participation and social media in samples that may have less access to after-school activities. Finally, the present research utilized self-report data which may not provide the full picture of youth experiences on social media or in after-school programming.

The present research also was conducted before social distancing and remote learning were introduced to schools and after-school programs as a result of the COVID-19 pandemic. During this time, school and after-school programming could have been provided entirely or partially in the digital context. This experience may have changed youth’s perception, understanding, and engagement in digital spaces, and is also necessary to document in future research.

Furthermore, questions remain about how to assess the rapidly changing digital context. Diverse methods including self-report, observed, and networked methods could be employed. More detailed information about the links between activity participation and social media engagement should be elucidated particularly in the unique cases when after-school activities make intentional efforts to support youth’s positive and informed engagement on social media. We hope that this research, though preliminary, provides inspiration for future research.

### Conclusion

Taken together, these findings indicated preliminary evidence of a link between after-school activity participation, in terms of friends from SM, and PYD-related social media use. Furthermore, these findings may provide after-school practitioners with ideas on how to develop programming that may provide social role modeling for how their youth connect to peers online. Peer relationship building is a natural part of after-school program environments. Knowing that youth in after-school programs may continue to connect with each other digitally outside of the program time and space may prompt program staff to be more intentional in talking about how (a) friendships carry over to different settings, (b) friends can support each other through different aspects of their lives, and (c) programs can infuse frequent opportunities for peer relationship building. We encourage after-school program practitioners to also take a PYD perspective on digital spaces, given the findings in the present research. They may continue to support these types of caring and connected behaviors online by sharing messaging about engaging with their friends from afterschool in supportive ways in and outside of the program. Future research may take up the unanswered questions that arise from these preliminary findings including what features of activity participation (e.g., adult supervision, hands-on learning, group activities) can shape online peer communities, and how activities and online behaviors can be explicitly linked to foster positive youth development.

## Figures and Tables

**Table 1.** Checking Social Media

	*M*/%	*SD*	*B*	*SE*	β	*p*	*R* ^2^	*F*
Outcome	3.40	1.91					0.41	33.49***
Black^a^	0.10	0.29	0.25	0.24	0.04	.303		
Latinx^a^	0.16	0.36	0.27	0.19	0.05	.167		
Asian^a^	0.08	0.28	−0.47	0.26	−0.07	.067		
Female^b^	0.53	0.50	0.19	0.14	0.05	.168		
Grade	7.06	0.81	0.06	0.09	0.03	.476		
Free/reduced-price lunch^c^	0.18	0.38	0.51	0.19	0.10	.006		
SM friend—same/younger classmates^d^	0.58	0.49	0.91	0.18	0.24	< .001		
SM friend—older classmates^d^	0.46	0.50	1.00	0.18	0.26	< .001		
SM friend—afterschool^d^	0.56	0.50	0.93	0.16	0.24	< .001		
Time in afterschool^e^	1.66	1.45	−0.03	0.05	−0.02	.519		

*Note*. *N* = 471. SM = social media.

a 0 = white, 1= Black, Latinx, or Asian.

b 0 = male, 1 = female.

c 0 = not reporting free/reduced lunch, 1 = reporting free/reduced-price lunch.

d 0 = not reporting this type of friend on social media, 1 = reporting this type of friend on social media.

e time reported in days attended.

*** *p* < .001

**Table 2.** Seeking Support

	*M*/%	*SD*	*B*	*SE*	β	*p*	*R* ^2^	*F*
Outcome	2.26	0.92					0.11	6.72***
Black^a^	0.10	0.30	0.10	0.14	0.03	.488		
Latinx^a^	0.15	0.36	0.16	0.12	0.06	.179		
Asian^a^	0.08	0.27	0.04	0.16	0.01	.797		
Female^b^	0.55	0.50	0.18	0.09	0.10	.033		
Grade	7.07	0.82	−0.04	0.05	−0.04	.415		
Free/reduced-price lunch^c^	0.18	0.39	0.29	0.11	0.12	.009		
SM friend—same/younger classmates^d^	0.59	0.49	0.11	0.11	0.06	.324		
SM friend—older classmates^d^	0.47	0.50	0.35	0.11	0.19	.001		
SM friend—afterschool^d^	0.58	0.49	0.27	0.10	0.14	.006		
Time in afterschool^e^	1.64	1.44	0.01	0.03	0.01	.812		

*Note*. *N* = 445. SM = social media.

a 0 = white, 1= Black, Latinx, or Asian.

b 0 = male, 1 = female.

c 0 = not reporting free/reduced lunch, 1 = reporting free/reduced lunch.

d 0 = not reporting this type of friend on social media, 1 = reporting this type of friend on social media.

e time reported in days attended.

*** *p* < .001

**Table 3.** Providing Support

	*M*/%	*SD*	*B*	*SE*	β	*p*	*R* ^2^	*F*
Outcome	3.31	1.14					0.19	11.82***
Black^a^	0.09	0.29	0.26	0.17	0.07	.125		
Latinx^a^	0.16	0.37	0.31	0.13	0.10	.022		
Asian^a^	0.08	0.28	0.06	0.18	0.02	.726		
Female^b^	0.53	0.50	0.49	0.10	0.22	< .001		
Grade	7.05	0.81	−0.13	0.06	−0.09	.034		
Free/reduced-price lunch^c^	0.18	0.38	−0.09	0.13	−0.03	.485		
SM friend—same/younger classmates^d^	0.57	0.50	0.45	0.13	0.19	.001		
SM friend—older classmates^d^	0.46	0.50	0.15	0.13	0.06	.257		
SM friend—afterschool^d^	0.56	0.50	0.38	0.11	0.17	.001		
Time in afterschool^e^	1.65	1.45	0.00	0.03	0.00	.920		

*Note*. *N* = 474. SM = social media.

a 0 = white, 1= Black, Latinx, or Asian.

b 0 = male, 1 = female.

c 0 = not reporting free/reduced lunch, 1 = reporting free/reduced lunch.

d 0 = not reporting this type of friend on social media, 1 = reporting this type of friend on social media.

e time reported in days attended.

*** *p* < .001

**Table 4.** Experiencing Negativity

	*M*/%	*SD*	*B*	*SE*	β	*p*	*R* ^2^	*F*
Outcome	1.49	0.64					0.03	2.53**
Black^a^	0.08	0.28	0.01	0.11	0.00	.943		
Latinx^a^	0.16	0.36	−0.02	0.09	−0.01	.812		
Asian^a^	0.09	0.29	−0.04	0.11	−0.02	.685		
Female^b^	0.54	0.50	0.06	0.06	0.05	.341		
Grade	7.11	0.80	0.05	0.04	0.07	.197		
Free/reduced lunch^c^	0.18	0.38	0.01	0.08	0.00	.948		
SM friend—same/younger classmates^d^	0.58	0.49	−0.01	0.08	0.00	.939		
SM friend—older classmates^d^	0.46	0.50	0.19	0.08	0.15	.020		
SM friend—afterschool^d^	0.57	0.50	0.09	0.07	0.07	.217		
Time in afterschool^e^	1.72	1.41	0.00	0.02	0.00	.960		

*Note*. *N* = 425. SM = social media.

a 0 = white, 1= Black, Latinx, or Asian.

b 0 = male, 1 = female.

c 0 = not reporting free/reduced lunch, 1 = reporting free/reduced lunch.

d 0 = not reporting this type of friend on social media, 1 = reporting this type of friend on social media.

e time reported in days attended.

** *p* = .006
